# Highly Sensitive Fluorescent Sensing for Nitrobenzene of Cd^II^ Complexes Based on Three Isomers and a Bis-Imidazole Ligand

**DOI:** 10.3390/molecules29112475

**Published:** 2024-05-24

**Authors:** Xue Yang, Wanting Liu, Yixia Ren, Xiufang Hou, Jinfeng Li

**Affiliations:** Laboratory of New Energy and New Function Materials, Shaanxi Key Laboratory of Chemical Reaction Engineering, College of Chemistry and Chemical Engineering, Yan’an University, Yan’an 716000, China; xueyang19961224@163.com (X.Y.); liuwanting0726@163.com (W.L.); houxiufang@yau.edu.cn (X.H.); lijinfengxiaofeng@163.com (J.L.)

**Keywords:** Cd (II) complex, crystal structure, fluorescent sensing properties, nitrobenzene

## Abstract

Detection of nitro pollutants is an important topic in environmental protection. A total of 3 Cd (II) complexes (**1**–**3**) based on 3 soft organic isomers, n-(3,5-dicarboxylato benzyloxy) benzoic acid (*n* = 2, 3 or 4-H_3_DBB), and a linear N-donor ligand, 3-bis(imidazole-l-ylmethyl) benzene (3-bibz), have been synthesized hydrothermally. Structural diversity of Complexes **1**–**3** displays the architectural 2D or 3D change: Complex **1** exhibits a 2D network featuring tri-nuclear metal units, Complex **2** is a 3D framework based on similar tri-nuclear metal units, and Complex **3** shows a 3D network with binuclear units. Fluorescent sensing properties exhibited in all these complexes have been discovered to detect nitrobenzene (NB) selectively and sensitively. In particular, Complex **3** possesses high sensitivity for NB with the lowest detection limit of 1.15 × 10^−10^ M. The results of the theoretical calculation verified the fluorescence detection mechanism of NB by these Cd-based complexes. Therefore, these Cd-based complexes might be used as excellent luminescent sensors for NB.

## 1. Introduction

The main use of nitrobenzene (NB) as a commonly used chemical substance is in the manufacture of aniline, which is commonly used as an insulating substance and a glossing agent [1]. In industry, the high use of NB can lead to human diseases and ecological damage due to environmental pollution [2]. Therefore, we need to carry out effective detection and treatment of NB to contribute to environmental protection. Currently, there are many methods for the detection of nitrobenzene, such as high-performance liquid chromatography (HPLC), ultraviolet–visible spectrophotometry (UV–vis), gas chromatography (GC), and gas chromatography–mass spectrometry (GC–MS) [3,4,5,6,7]. However, these methods are not convenient to carry out due to the high requirements of instrumentation, harsh testing conditions, etc. Therefore, we need an assay that can be fast, easy, and accurate to achieve this goal, such as a fluorescence sensing detection method.

Excellent fluorescence sensing materials are the key to the detection of nitrobenzene. Luminescent metal-organic coordination polymer is an effective and convenient class of fluorescent sensing materials [8]. More and more of such materials are found to be capable of effective fluorescence sensing of heavy metals, industrial pollutants, pesticides, etc., with excellent sensing selectivity, sensitivity, and stability [9,10,11,12]. Among them, Cd-based coordination polymers showed good fluorescence sensing properties for different analysts, such as metal ions, anions, nitro-compounds, and so on [13]. For example, an aminoisophthalate bridged Cd(II)-2D coordination polymer has been reported to selectively detect Pd^2+^ in an aqueous medium with a limit of detection (LOD) of 0.08 μM. It has also been found that the fluorescence quenching by Pd^2+^ might be caused by the formation of a coordinated –COO–Pd bond [14]. Compared with rare earth elements, Cd-based coordination polymers have their advantages due to their shorter wavelength luminescence, wide luminescence range, and stable luminescence performance, so Cd-based coordination polymers are chosen as the target complexes. Tricarboxylic acid ligand has three carboxylic acid groups, multiple coordination sites, and rich coordination modes, facilitating the construction of a structurally rich complex. The bridging nitrogen-containing auxiliary ligands are used widely to link the inorganic building units with various structural characteristics. The above studies have shown that Cd-based coordination polymers can be used as an effective material for fluorescence sensing.

Herein, we designed and synthesized three cadmium coordination polymers with three soft tri-carboxylate isomers (H_3_DBB) and one linear N-donor ligand (3-bibz) hydrothermally (Figure S3). Their crystal structures, IR spectra, and thermal stability were investigated. Their excellent and stable fluorescent performance inspires us to deeply study their sensing properties, and the results show all complexes could detect slight nitrobenzene in an aqueous solution with low detection limits, especially Complex **3**, which has the lowest detection limit among these complexes. The mechanism of fluorescence sensing for nitrobenzene was verified by theoretical calculation. Complexes **1**–**3** are very promising as effective sensors for nitrobenzene.

## 2. Results and Discussion

### 2.1. Crystal Structures

**Figure 1:**
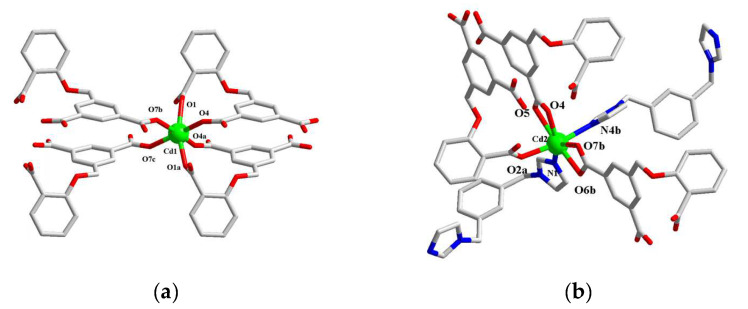

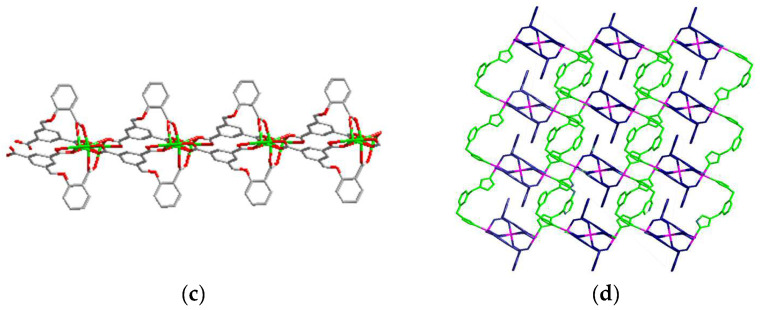
Coordination environment of Cd1 ion (**a**) and Cd2 ion (**b**) in Complex **1**; (**c**) one-dimensional chain structure (along b axis); (**d**) three-dimensional supramolecular framework of Complex **1**.

**[Cd_1.5_(2-DBB)(3-bibz)](1).** Single crystal X-ray diffraction analysis shows that the central Cd1 ion is coordinated with the six oxygen atoms of the four 2-DBB^3−^ ligands (O1, O1a, O4, O4a, O7b, and O7c) to form an octahedral configuration with O1A, O1B, O4, and O7 as the plane and O4 and O7 as the vertices on both sides (Figure 1a). The central ion Cd2 is coordinated with five oxygen atoms (O2a, O4, O5, O6b, and O7b) of three 2-DBB^3−^ ligands, and is coordinated with two nitrogen atoms (N1, N4b) of two 3-bibz ligands. A pentagonal bipyramid configuration with O5, O4, N1, O7b, and O6b as the plane and N4b and O2 as the vertices is formed (Figure 1b). The bond length range of Cd-O is 2.201–2.569 Å, the bond length range of Cd-N is 2.265 and 2.302 Å, the bond angle range of O-Cd-O is 52.5–175.16°, and the bond angle range of N-Cd-O is 85.51–171.1°.

One Cd1 ion and two Cd2 ions form a [Cd_3_(COO)_4_]^2−^ tri-nuclear metal unit through the bridging of the carboxyl group of the 2-DBB^3−^ ligand (the distance of the adjacent Cd ions is 3.565 Å). This tri-nuclear metal unit forms a one-dimensional chain structure along the b-axis through the spacers of 2-DBB^3−^ in (κ_1_)-(κ_1_)-(κ_1_):μ_3_ coordination mode (Chart S1a and Figure 1c). The one-dimensional chains are then expanded into a two-dimensional network structure through the bridging of 3-bibz ligands (Figure 1d). Adjacent two-dimensional networks form a partially interspersed three-dimensional supramolecular structure through many weak hydrogen bonds (C8-H8B···C24: 3.414 Å; C24-H24···O6: 3.403 Å; C27-H27···O3: 3.382 Å; C28-H28···O5: 3.216 Å; O6-H6···C26: 3.383 Å) among the 2-DBB^3−^ and 3-bibz ligands from adjacent different two-dimensional networks.

**Figure 2:**
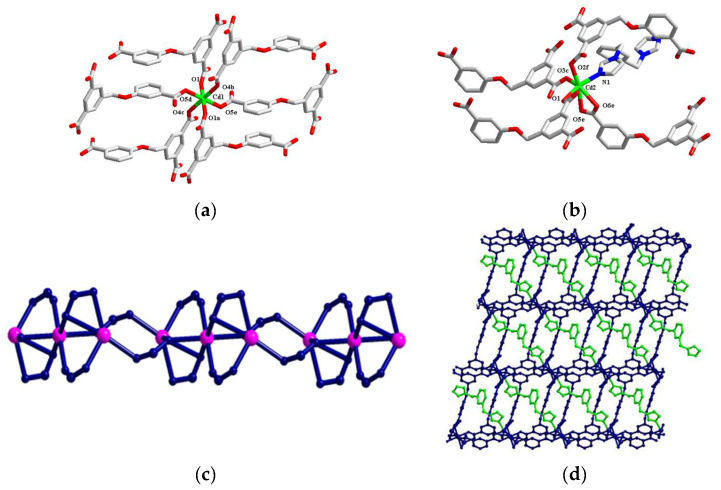
Coordination environment of the central ions Cd1 (**a**) and Cd2 (**b**) in Complex **2**; (**c**) one-dimensional chain structure; (**d**) three-dimensional structure.

**[Cd_3_(3-DBB)_2_(3-bibz)](2)**. Complex **2** is a three-dimensional Cd (II) organic framework based on tri-nuclear metal units. There are two crystallographically independent Cd^2+^ ions in Complex **2**. Among them, Cd1 is connected with six oxygen ions (O1, O1a, O4b, O4c, O5d, and O5e) of six 3-DBB^3−^ ligands to form an apex with O4b and O4c, and O1, O1a, O5d, and O5e as a planar six-coordinate octahedral configuration (Figure 2a). And Cd2 is connected to five oxygen ions (O1, O2f, O3c, O5e, and O6e) of five 3-DBB^3−^ ligands, and is connected to a nitrogen atom (N1) of a 3-bibz ligand. Forming a structure with O1 and N1 as the vertex is a six-coordinate octahedral configuration with O2f, O3c, O5e, and O6e as the plane (Figure 2b). The bond length range of Cd-O is 2.179–2.596 Å, the bond length of Cd-N is 2.223 Å, the bond angle range of O-Cd-O is 50.80–180°, and the bond angle range of O-Cd-N is 79.85–158.15°. Similarly, one Cd1 and two Cd2 ions form a tri-nuclear metal unit [Cd_3_(COO)_4_]^2−^ through the bridging of the carboxyl group of the 3-DBB^3−^ ligand, where the intermetallic distance of Cd ions is 3.445 Å. The tri-nuclear metal unit is connected by the carboxyl oxygen atom of 3-DBB^3−^ to form a 1D chain structure along the a-axis (Figure 2c). The coordination mode of 3-DBB^3−^ in the complex is (κ_1_)-(κ_1_)-(κ_1_):μ_3_, which further extends 1D chains to form a 2D network in the ab plane (Figure 2d). The 3-bibz ligand connects two Cd2 central atoms through the bridge of the terminal nitrogen atom and intersperses in the 3D pores to make the structure more stable.

**Figure 3:**
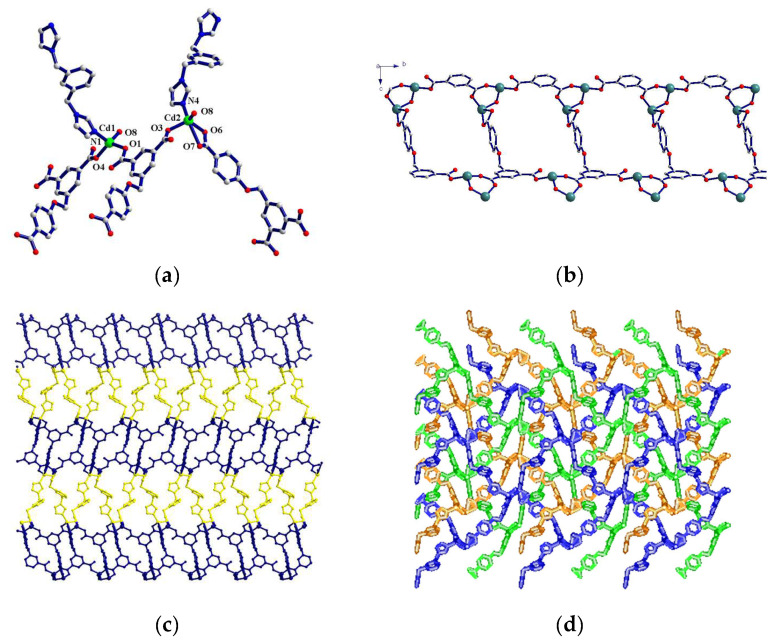
(**a**) Coordination environment of the central ions Cd1 and Cd2 in Complex **3**; (**b**) one-dimensional chain structure based on 4-DBB^3−^ ligand along b-axis; (**c**) two-dimensional network; (**d**) the triple interspersed structure.

**[Cd_2_(4-DBB)(OH)(3-bibz)](3)**. Structural analysis of Complex **3** shows that the central ion Cd1 is coordinated with two oxygen atoms (O1, O4) from two 4-DBB^3−^ ligands, one nitrogen atom (N1) of 3-bibz, and an oxygen atom (O8) of a hydroxyl group to form a tetra-coordinated tetrahedral configuration. The central ion Cd2 ion is surrounded by three oxygen atoms (O3, O6, and O7) of the two 4-DBB^3−^ ligands, a nitrogen atom (N4) of the 3-bibz ligand, and an oxygen atom (O8) of a hydroxyl group to form a five-coordinate pentahedral configuration (Figure 3a). The bond length range of Cd-O is 1.971–12.64 Å, the bond length range of Cd-N is 2.015–2.038 Å, and the bond angle scope of O/N-Cd-O/N is 49.2–150.8°. Cd1 and Cd2 are linked by one hydroxyl group and one bridged carboxyl group of a 4-DBB^3−^ ligand to form a binuclear unit. The binuclear units are connected by the carboxyl groups of three 4-DBB^3−^ ligands in the coordinated mode of (κ_1_)-(κ_1_)-(κ_1_-κ_1_):μ_4_ to produce a 1D ladder-like chain structure (Figure 3b). The 1D ladder-like chains are connected by 3-bibz ligands to form a 2D network structure. The 1D ladder chain forms a 2D wavy structure at a corner of about 80° through a flexible bridge link of the 4-DBB^3−^ ligand (Figure 3c). The 3-bibz ligand forms pillars between the 2D wave structures in a two-tooth bridge coupling mode, further forming the 3D microporous skeleton structure. In this coordination polymer, the micropore structure is disrupted by the three identical 3D skeletons interspersed, forming a dense triple-interspersed structure (Figure 3d).

### 2.2. Structural Discussion

Structural diversity of Complexes **1**–**3** exhibits the change from 2D network (**1**) to 3D frameworks (**2** and **3**) mainly due to the coordination environment difference of Cd ions and the diversified coordination fashions of three isomers *n*-H_3_DBB. In Complexes **1**–**3**, Cd ions lie in a variety of coordination polyhedron environments and the coordination numbers range from four to seven, exhibiting tetrahedron, pentagonal bipyramid, and octahedral geometries. Three isomers of n-H_3_DBB ligands as μ_3_- or μ_4_-connected notes adopt three high-coordination fashions to link Cd ions (Chart S1). The N-containing auxiliary ligands (3-bibz) in these complexes act as the bridging ligands to reinforce or construct high-dimensional networks.

### 2.3. Thermogravimetric Analysis

To investigate the thermal stability of Complexes **1**–**3**, the TG curves were plotted, as shown in Figure S1. The thermal analysis process of Coordination Polymer **1** is performed step by step: firstly, at 30 °C to 226 °C, the stability of the coordination polymer is general. The weight loss starts from heating and the weight loss rate is 9.17%, probably due to the partial removal of ligands. Then, there is a large weight loss process until 868 °C. The weight loss rate is 54.02% at this stage since the skeleton of 1 breaks. The thermal decomposition process of Complex **2** is completed in two steps, similar to that of Complex **1**: (1) from the beginning of heating, a weight loss takes place, and (2) from 156 °C to 865 °C, Complex **2** has a large weight loss process, meaning the skeleton collapse of Complex **2** with the weight loss rate is 56.66% in this stage. The thermal decomposition process of the Coordination Polymer **3** also requires 2 steps: from 30 °C to 118 °C is a small weight loss process with a weight loss rate of 7.2%; then, starting from 118 °C, Complex **3** begins the skeleton collapse until 863 °C with the weight loss rate of 57.07% at this stage.

### 2.4. Luminescent Properties

The excitation and emission spectra of Complexes **1**–**3** were investigated and showed good luminescence in solid-state or solution. Coordination polymers have the strongest emission peaks at 435 nm (λ_ex_ = 318 nm) for Complex **1**, 418 nm (λ_ex_ = 345 nm) for Complex **2**, and 424 nm (λ_ex_ = 350 nm) for Complex **3** (Figure S2a). In comparison with the fluorescence properties of free organic ligands H_3_DBB and 3-bibz, the luminescence of these complexes can be attributed to the π*→π transition of the ligands. Whereas the fluorescence emission of these complexes in aqueous solution is shown in Figure S2b, the positions of the emission peaks of all three complexes are blue-shifted at 416 nm (λ_ex_ = 303 nm), 348 nm (λ_ex_ = 287 nm), and 375 nm (λ_ex_ =275 nm), respectively, which may be attributed to solvent effect [15]. The good fluorescence emission of Cd-based coordination polymers is suitable for fluorescence sensing applications.

### 2.5. Fluorescence Detection of NB

In order to investigate the potential fluorescence properties, we carried out their fluorescence sensing performances and discovered that all coordination polymers exhibited excellent fluorescence quenching on NB with high selectivity and sensitivity. The mixture of powder (3 mg) and water (10 mL) of Coordination Polymers **1**–**3** was sonicated for 1 h by the ultrasonic method to form an aqueous suspension and then left to stand for 24 h. Organic solvent (20 μL) was added to a Cd-CPs aqueous solution (1 mL), respectively, and the fluorescence intensity was measured at room temperature by an F-7100 fluorescence spectrophotometer (Hitachi Corporation, Tokyo, Japan). The fluorescence intensity was compared with those of the blank Cd-CPs aqueous solution, and the fluorescence effect of different solvents on the complex was concluded.

**Figure 4:**
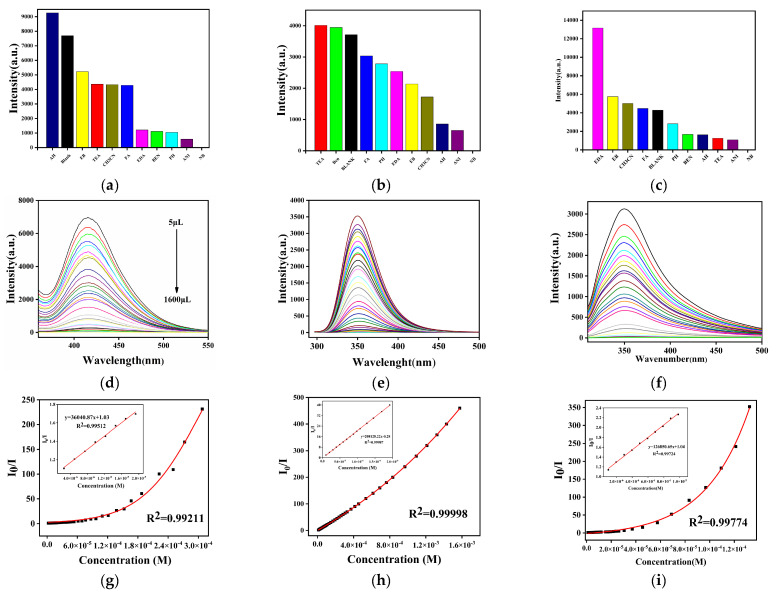
Qualitative selection diagram for NB of (**a**) **1**, (**b**) **2**, and (**c**) **3**; quantitative analysis diagram of (**d**) **1**, (**e**) **2**, and (**f**) **3**; linear relationship diagram of (**g**) **1**, (**h**) **2**, and (**i**) **3**.

The selective analysis of Coordination Polymers **1**–**3** showed that all of them had a complete quenching effect on NB, respectively, and we conducted in-depth research on them (Figure 4a–c). The UV–vis absorption spectrum of NB is shown in Figure S4. Then, we took the suspension (1 mL) of coordination polymers, added 0.5 mmol·L^−1^ of NB, and investigated the changes in fluorescence intensity, respectively. As the volume of the NB solution increases, the fluorescence emission intensity gradually decreases (Figure 4d–f). For Complex **1**, the whole titration region is consistent with the exponential function, but it exhibits good linearity in the low concentration range as *I*_0_/*I* = 36040.87[NB] + 1.03 (*I*_0_ and *I* represent the luminescence intensity of aqueous suspension of the coordination polymer before and after exposure to NB concentration, respectively) with good linearity of *R*^2^ = 0.9921 and a low detection limit (LOD) of 4.42 × 10^−7^ mol·L^−1^ (Figure 4g). The selectivity analysis of Coordination Polymers **2** and **3** also exhibited an excellent fluorescence quenching on NB with high selectivity and sensitivity, as shown in Figure 4h,i. Especially for Coordination Polymer **3**, the highly sensitive detection for NB is with the lowest detection limit of 1.15 × 10^−10^ mol·L^−1^ compared with the reported similar MOFs [16], which might mean Coordination Polymer **3** will be an excellent sensor for trace detection of NB in an aqueous solution in the future. We further investigated the fluorescence response of the three complexes to an electron-withdrawing solvent (such as CH_3_CN) and other nitro compounds, as well as their interference with nitrobenzene (Figure S5). It can be clearly seen from the experiment that their fluorescence quenching effect on Cd-CPs **1**–**3** is not as good as those of nitrobenzene, and there is no interference with the fluorescence sensing on NB when they exist.

### 2.6. Nitrobenzene Fluorescence Quenching Theory

**Figure 5:**
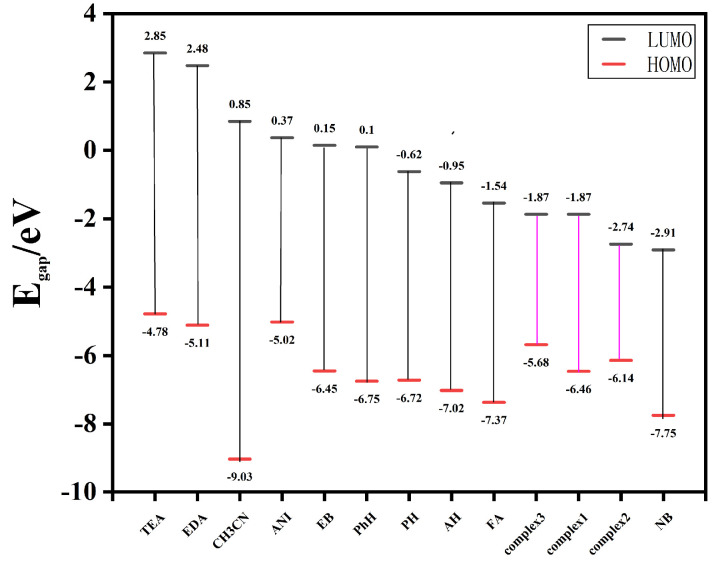
HOMO and LUMO orbital energies of three complexes (**1**–**3**) and organic solvents.

By comparing the theoretical simulated patterns of Complexes **1**–**3**, the measured values, and those treated with NB, all the framework of the complexes did not change (Figure S6), meaning skeleton collapse is not the primary cause of fluorescence bursts. To explain the mechanism of fluorescence quenching by nitrobenzene for Complexes **1**–**3**, the orbital energy levels (HOMO/LUMO) of various organic solvents and Coordination Polymers **1**–**3** are calculated by using the density functional theory. We have optimized the molecular structures by choosing the general function B3LYP and the basis group 6–31 g/LANL2DZ and analyzed the nature of the HOMO/LUMO orbitals. The fluorescence detection mechanism of Coordination Polymers **1**–**3** against nitrobenzene was investigated by calculating the energies of the HOMO/LUMO orbitals According to the calculated results, we found that the LUMO levels of all organic solvents except NB are higher than those of Coordination Polymers **1**–**3** (Figure 5). The lower the LUMO energy of the analyte, the easier the transition of the excited state electrons. Therefore, under light-induced conditions and concerning electron transfer to NB, it is believed that the light-induced electron transfer (PET) effect is responsible for the quenching of nitrobenzene fluorescence.

## 3. Materials and Methods

### 3.1. Experimental Materials and Instrument

All solvents and reagents are available and used as required. The thermogravimetric analysis adopts a NETZSCH STA 449C micro-analyzer, heated in an air atmosphere from 30 °C to 900 °C. Use a Flash2000 organic element (Thermo Fisher Scientific, Waltham, MA, USA) analyzer for elemental analysis of C, N, H, and O elements. The fluorescence spectrum was measured at room temperature using an F-7100 fluorescence spectrophotometer. A Rigaku Ultima IV diffractometer was used to obtain the powder X-ray diffraction patterns (PXRD). The infrared spectrometer was determined by an Affinity-1 infrared spectrometer (Shimadzu Company, Kyoto, Japan).

### 3.2. Crystal Preparation of [Cd_1.5_(2-DBB)(3-bibz)] **(1)**, [Cd_3_(3-DBB)_2_(3-bibz)] **(2)** and [Cd_2_(4-DBB)(OH)(3-bibz)]**(3)**

The mixture of cadmium nitrate (0.031 g), n-H_3_DBB (0.016 g) (2-H_3_DBB for Complex **1**, or 3-H_3_DBB for Complex **2**, or 4-H_3_DBB for Complex **3**), and 3-bibz (0.015 g) in different solvent conditions (3 ml of water and 1 ml of methanol for Complex **1**, 2 mL of water and 2 ml of isopropanol for Complex **2**, and 3 mL of water for Complex **3**), as well as NaAc (0.078 g), was put into 25 mL of Teflon in a stainless steel container lined with dragon, heated in a high-temperature oven at 160 °C for 72 h, and then the mixture was slowly cooled to room temperature to obtain colorless rod-shaped crystals (**1**), pale yellow block crystals (**2**), and yellow nubbly crystals (**3**). These crystals were washed with ethanol and water and collected after drying. Anal. calcd for C_30_H_23_Cd_1.5_N_4_O_7_ (**1**) (%): C, 50.03; H, 3.22; N, 7.78. Found(%): C, 50.34; H, 3.31; N, 7.49. IR(KBr, cm^−1^): 3404 (m), 3080 (w), 1577 (s), 1442 (w),1384 (s), 1286 (w), 1224 (m). Anal. calcd for C_46_H_27_Cd_3_N_4_O_14_ (**2**) (%): C, 46.16; H, 2.27; N, 4.68. Found(%): C, 46.32; H, 2.51; N, 4.92. IR(KBr, cm^−1^): 3116 (w), 3072 (w), 1554 (m), 1440 (w), 1363 (s), 1226 (m). Anal. calcd for C_30_H_24_Cd_2_N_4_O_8_ (**3**) (%): C, 45.42; H, 3.05; N, 7.06; Found(%): C, 45.66; H, 3.21; N, 6.94. IR(KBr, cm^−1^): 3427 (m), 3126 (m), 1610 (s), 1381 (s), 1236 (m).

### 3.3. Crystal Measurement

The suitable crystals of Complexes **1**–**3** have been chosen and performed on the CCD diffractometer for X-ray crystallographic analysis. During data collection, the crystal was kept at 296(2)K. Using Olex2, the structures are solved by using the ShelXT structural solution of the inherent phase and refined using the ShelXL optimization package of least square optimization [17,18]. The crystallographic data of Cd-based Complexes **1**–**3** and the selected bond lengths and bond angles are listed in Table S1, Table S2(**1**), Table S3(**2**), and Table S4(**3**), respectively. CCDC numbers: 2343262(**1**), 2343263(**2**), and 2343264(**3**).

### 3.4. Fluorescence Sensing Experiment

Take the MOF powder (3 mg) and add (10 mL) of distilled water, sonicate the MOF-containing aqueous solution (1 mL) for 1 h, let it stand for 24 h, take the suspension, add 1 mL of different pure organic solvents, shake well, and perform fluorescence research. Organic solvents include ethanediamine (EDA), acetonitrile (CH_3_CN), ethylbenzene (EB), ethyl alcohol (MtOH), Benzene (BEN), acetaldehyde (AH), triethylamine (TEA), formaldehyde (FA), Propionaldehyde (PH), acetone (CP), phenylamine (ANI), and nitrobenzene (NB).

## 4. Conclusions

Using three isomers and a bis-imidazole ligand, three Cd-based coordination polymers **1**–**3** have been successfully designed and synthesized, exhibiting two- or three-dimensional structural features with di- or tri-nuclear inorganic units. Fluorescence sensing of solvent molecules showed that NB had a fluorescence quenching effect on all of these complexes, especially for Complex **3** with a detection limit exceeding the ppb level. We further investigated the mechanism of NB bursting by the complexes through theoretical calculations and found that it can be attributed to the photoinduced electron transfer mechanism. This work provides new ideas for the development of efficient NB sensing materials.

## Data Availability

Data are contained within the article and Supplementary Materials.

## Supplementary Materials

The following supporting information can be downloaded at https://www.mdpi.com/article/10.3390/molecules29112475/s1, Chart S1: The coordination modes of n-(3,5-dicarboxylato benzyloxy) benzoic acids; Figure S1: TG curves for complexes **1**–**3**; Figure S2: The emission spectra of the coordination polymers **1**–**3**; Figure S3: (a–c) The structures of ligands [n-(3,5-dicarboxylato benzyloxy) benzoic acid (n = 2, 3 or 4-H3DBB) and (d) a linear N-donor ligand: 3-bis(imidazole-1-ylmethyl) benzene (3-bibz)]; Figure S4: The UV-vis absorption spectrum of nitrobenzene; Figure S5: Fluorescence sensing characterization of complexes **1**–**3** for nitro explosives (a-c); Anti-jamming experiment of p-nitrobenzene (b–f).; Figure S6: Theoretical and experimental values of complexes **1**–**3** (a–c) and PXR D patterns of soaked NB; Table S1: Crystal data and structure refinement details for **1**–**3**; Table S2: Selected bond lengths and bond angles of **1**–**3**; Table S3: Selected bond lengths (Å) and bond angles (°) of **2**; Table S4: Selected bond lengths (Å) and bond angles (°) of **3**.
