# Supplementary material for: Highly Sensitive Fluorescent Sensing for Nitrobenzene of CdII Complexes Based on Three Isomers and a Bis-Imidazole Ligand
Source: Molecules. 2024 May 24;29(11):2475. doi: 10.3390/molecules29112475 (PMC11173416; doi:10.3390/molecules29112475)
Supplement: Supplementary file 1 [file molecules-29-02475-s001.zip › molecules-3013983-supplementary.pdf]

Highly sensitive fluorescent sensing for nitrobenzene of Cd^{II} complexes based on three isomers and a bis-imidazole ligand

Xue Yang[1], Wanting Liu[1], Yixia Ren,* Xiufang Hou, Jinfeng Li

College of Chemistry and Chemical Engineering, Laboratory of New Energy and New Function Materials, Shaanxi Key Laboratory of Chemical Reaction Engineering, Yan'an University, Yan'an 716000, P. R. China

For

Molecules

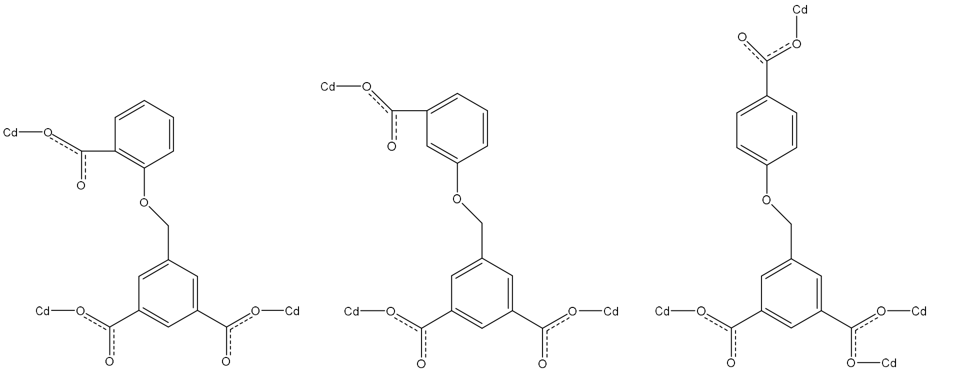

(a) (κ_1)-(κ_1)-(κ_1):μ_3 (b) (κ_1)-(κ_1)-(κ_1):μ_3 (c) (κ_1)-(κ_1)-(κ_1-κ_1):μ_4

Chart S1 The coordination modes of *n*-(3,5-dicarboxylato benzyloxy) benzoic acids (n = 2, 3 or 4-H_3DBB)

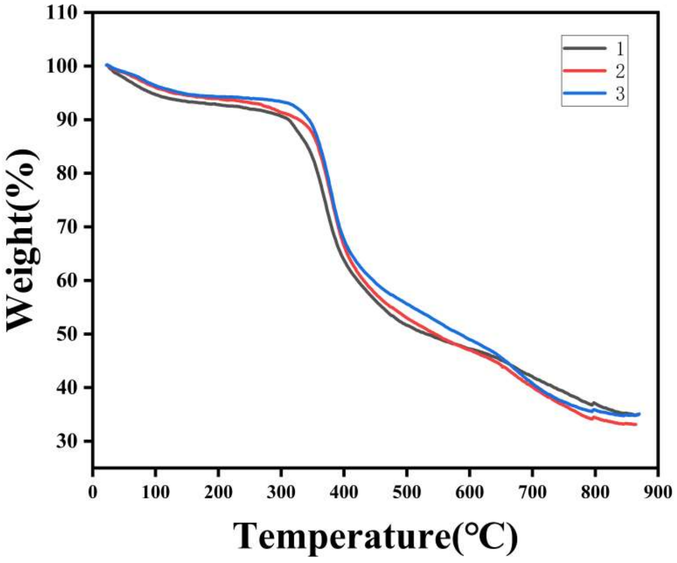

Figure S1 TG curves for complexes 1-3.

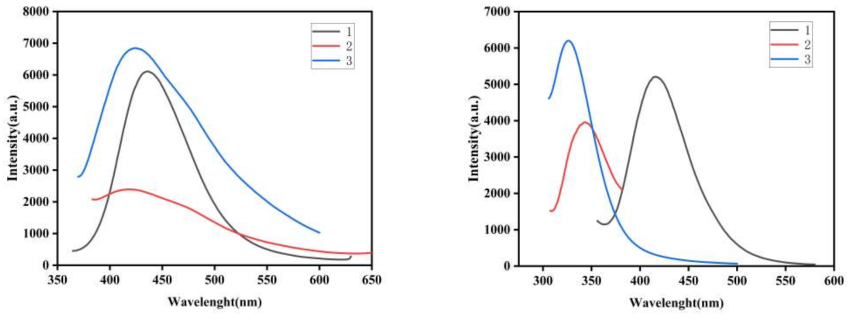

(a) (b)

Figure S2 The emission spectra of the coordination polymers **1-3** in solid state(a) and solution (b).

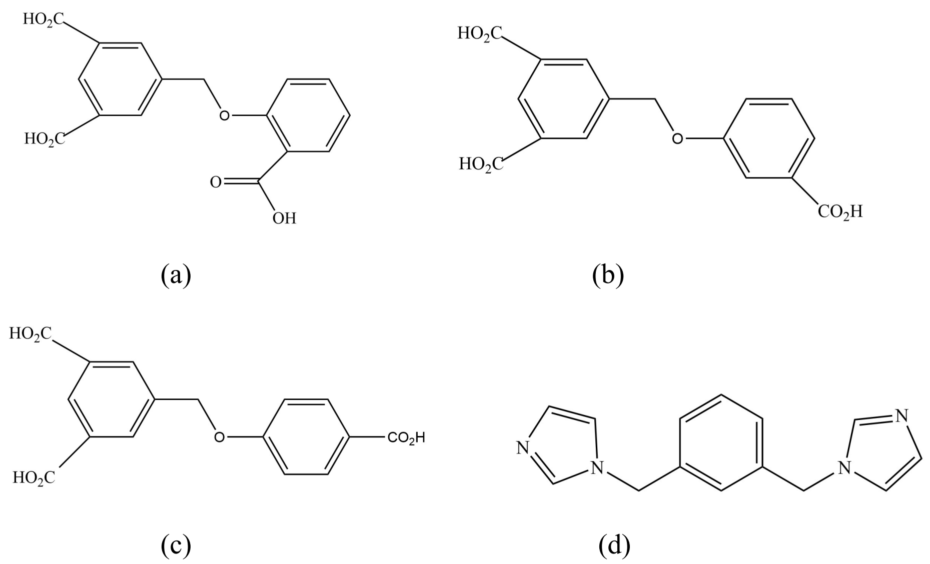

Figure S3 The structures of ligands [n-(3,5-dicarboxylato benzyloxy) benzoic acid (n = 2, 3 or 4-H3DBB) (a-c) and a linear N-donor ligand: 3-bis(imidazole-1-ylmethyl) benzene (3-bibz)] (d)

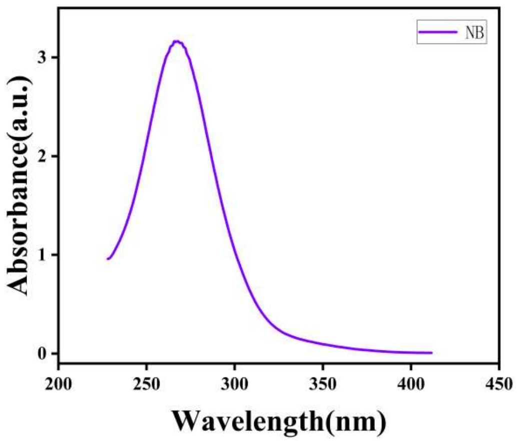

Figure S4 The UV-vis absorption spectrum of nitrobenzene

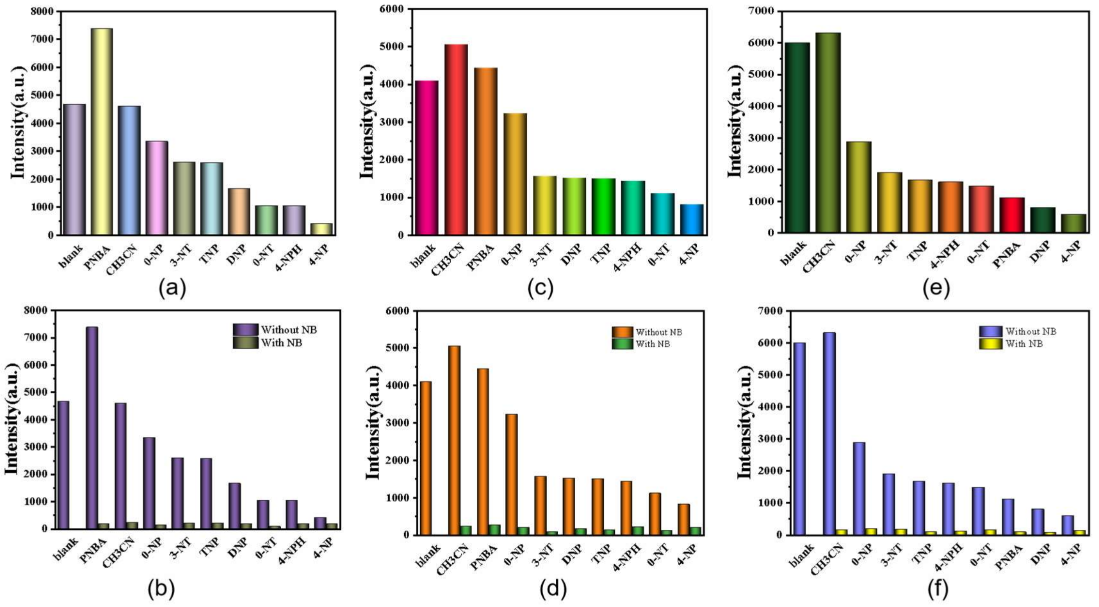

Figure S5 Fluorescence sensing characterization of complexes 1-3 for nitro explosives (a-c); Anti-jamming experiment of p-nitrobenzene (b-f).

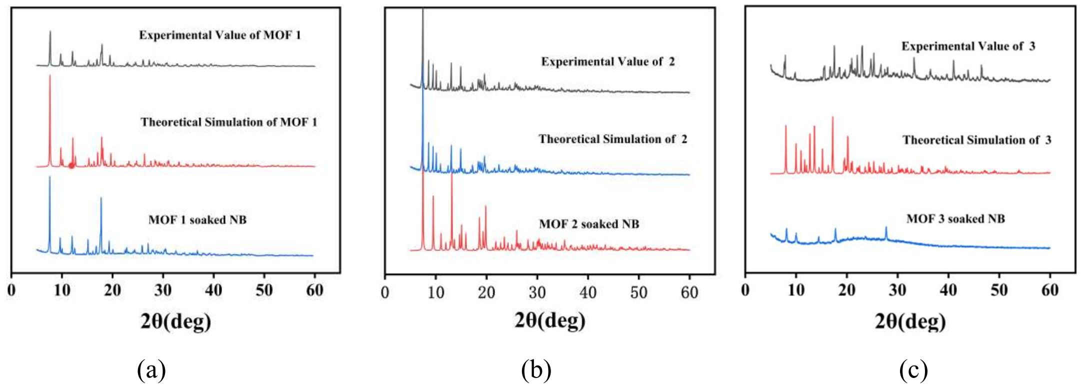

Figure S6 Theoretical and experimental values of complexes 1-3 (a-c) and PXRD patterns of soaked NB

Table S1 Crystal data and structure refinement details for **1-3**

**complexes 1-3**	**1**	**2**	**3**
Formula	$C_{30}H_{23}Cd_{1.50}N_4O_7$	$C_{46}H_{27}Cd_3N_4O_{14}$	$C_{30}H_{24}Cd_2N_4O_8$
Formula weight	720.12	1196.91	793.35
Crystal system	Monoclinic	Triclinic	Orthorhombic
Space group	*C*2/c	*P*-1	*P*na2$_1$
a (Å)	31.351(18)	9.3533(16)	14.836(6))
b (Å)	9.891(6)	9.7956(18)	9.759(4)

c (Å)	23.440(14)	12.0920(18)	22.293(9)
α (°)	90	99.495(4)	90
β (°)	131.531(7)	92.055(6)	90
γ (°)	90	103.023(6)	90
V (cm^{-3})	5441(6)	1061.5(3)	3228(2)
ρ (g cm^{-3})	1.758	1.872	1.633
Z	8	1	4
F(000)	2872	587	1568.0
Goof			
R_1,wR_2(I> 2sigmal(I))	R_1 = 0.0500, wR_2 = 0.1367	R_1 = 0.0515, wR_2 = 0.1039	R_1 = 0.0645, wR_2 = 0.1837
R_1,wR_2 (all data)	R_1 = 0.1561, wR_2 = 0.2098	R_1 = 0.1041, wR_2 = 0.1198	R_1 = 0.4968, wR_2 = 0.5858

$^aR_1 = \sum(|F_o|-|F_c|)/\sum|F_o|$; $^bwR_2 = \{\sum[w(|F_o|^2-|F_c|^2)^2]/\sum[w(|F_o|^2)^2]\}^{1/2}$.

Table S2 Selected bond lengths (Å) and bond angles (°) of **1**

bond lengths	Å	bond lengths	Å	bond lengths	Å
Cd(1)-O(1)	2.201(5)	Cd(2)-N(1)	2.265(6)	Cd(1)-O(4)	2.335(5)
Cd(1)-O(1)#1	2.201(5)	Cd(2)-O(2)#1	2.279(5)	Cd(2)-O(4)	2.312(5)
Cd(1)-O(7)#2	2.290(6)	Cd(2)-N(4)#4	2.302(6)	Cd(2)-O(5)	2.569(7)
Cd(1)-O(7)#3	2.290(6)	Cd(2)-O(6)#2	2.333(7)	Cd(2)-O(7)#2	2.563(6)
Cd(1)-O(4)#1	2.335(5)				
**bond angles**	**°**	**bond angles**	**°**	**bond angles**	**°**
N(4)#4-Cd(2)-O(6)#2	89.7(2)	O(1)-Cd(1)-O(1)#1	163.6(4)	O(7)#3-Cd(1)-O(4)	167.97(19)
O(4)-Cd(2)-O(6)#2	123.74(19)	O(1)-Cd(1)-O(7)#2	91.4(2)	O(4)#1-Cd(1)-O(4)	114.7(3)
N(1)-Cd(2)-O(7)#2	142.1(2)	O(1)#1-Cd(1)-O(7)#2	99.8(2)	N(1)-Cd(2)-O(2)#1	89.4(2)
O(2)#1-Cd(2)-O(7)#2	99.17(19)	O(1)-Cd(1)-O(7)#3	99.8(2)	N(1)-Cd(2)-N(4)#4	91.5(2)
N(4)#4-Cd(2)-O(7)#2	85.51(19)	O(1)#1-Cd(1)-O(7)#3	91.4(2)	O(2)#1-Cd(2)-N(4)#4	171.1(3)
O(4)-Cd(2)-O(7)#2	71.3(2)	O(7)#2-Cd(1)-O(7)#3	93.8(3)	N(1)-Cd(2)-O(4)	146.4(2)
O(6)#2-Cd(2)-O(7)#2	52.50(19)	O(1)-Cd(1)-O(4)#1	84.1(2)	O(2)#1-Cd(2)-O(4)	85.46(19)
N(1)-Cd(2)-O(5)	93.5(2)	O(1)#1-Cd(1)-O(4)#1	87.1(2)	N(4)#4-Cd(2)-O(4)	88.90(19)
O(2)#1-Cd(2)-O(5)	84.4(2)	O(7)#2-Cd(1)-O(4)#1	167.97(19)	N(1)-Cd(2)-O(6)#2	89.8(2)
N(4)#4-Cd(2)-O(5)	86.7(2)	O(7)#3-Cd(1)-O(4)#1	76.1(2)	O(2)#1-Cd(2)-O(6)#2	99.1(2)
O(4)-Cd(2)-O(5)	52.98(17)	O(1)-Cd(1)-O(4)	87.1(2)	O(7)#2-Cd(2)-O(5)	123.84(18)
O(6)#2-Cd(2)-O(5)	175.16(16)	O(1)#1-Cd(1)-O(4)	84.1(2)	O(7)#2-Cd(1)-O(4)	76.1(2)

Symmetry codes:#1 -x+2,y,-z+1/2; #2 x,y-1,z; #3 -x+2,y-1,-z+1/2; #4 x,-y,z-1/2.

Table S3 Selected bond lengths (Å) and bond angles (°) of **2**

bond lengths	Å	bond lengths	Å	bond lengths	Å
Cd(1)-O(1)#1	2.407(4)	Cd(1)-O(4)#3	2.179(4)	Cd(2)-O(1)	2.235(4)
Cd(1)-O(1)	2.407(4)	Cd(1)-O(5)#4	2.251(4)	Cd(2)-O(2)#6	2.463(5)

Cd(1)-O(4)#2	2.179(4)	Cd(2)-O(5)#5	2.416(4)	Cd(2)-O(3)#3	2.596(4)
bond angles	°	bond angles	°	bond angles	°
O(1)-Cd(2)-O(3)#3	107.07(15)	Cd(2)-Cd(1)-Cd(2)#1	180.0	O(4)#2-Cd(1)-O(1)#1	84.66(15)
O(6)#5-Cd(2)-Cd(1)	77.54(11)	O(1)-Cd(1)-Cd(2)#1	139.80(9)	O(4)#2-Cd(1)-O(4)#3	180.0(3)
O(6)#5-Cd(2)-O(5)#5	50.80(14)	O(1)#1-Cd(1)-Cd(2)#1	40.20(9)	O(4)#3-Cd(1)-O(5)#4	90.06(15)
O(2)#6-Cd(2)-Cd(1)	128.18(9)	O(1)#1-Cd(1)-Cd(2)	139.80(9)	O(4)#3-Cd(1)-O(5)#5	89.94(15)
O(2)#6-Cd(2)-O(6)#5	136.48(15)	O(1)-Cd(1)-Cd(2)	40.20(9)	O(4)#2-Cd(1)-O(5)#4	89.94(15)
O(2)#6-Cd(2)-O(5)#5	168.75(13)	O(1)#1-Cd(1)-O(1)	180.0	O(4)#2-Cd(1)-O(5)#5	90.06(15)
O(5)#5-Cd(2)-Cd(1)	40.80(9)	O(4)#2-Cd(1)-Cd(2)#1	66.43(12)	O(5)#4-Cd(1)-Cd(2)	131.11(11)
O(4)#3-Cd(1)-Cd(2)	66.43(12)	O(4)#2-Cd(1)-Cd(2)	113.57(12)	O(4)#3-Cd(1)-O(1)	84.66(15)
O(4)#3-Cd(1)-Cd(2)#1	113.57(12)	O(4)#3-Cd(1)-O(1)#1	95.34(15)	O(4)#2-Cd(1)-O(1)	95.34(15)

Symmetry codes:#1 -x+1,-y+1,-z; #2 -x+1,-y,-z; #3 x,y+1,z; #4 -x+1,-y+1,-z+1; #5 x,y,z-1; #6 -x+2,-y+1,-z

Table S4 Selected bond lengths (Å) and bond angles (°) of **3**

bond lengths	Å	bond lengths	Å	bond lengths	Å
Cd1-O8	1.971(7)	Cd1-N1	2.015(9)	Cd2-O8#1	1.932(7)
Cd1-O1	1.977(5)	Cd2-O6#2	2.049(8)	Cd2-O3	2.041(6)
Cd1-O4#1	1.999(5)	Cd2-O7#2	2.19(5)	Cd2-N4	2.038(6)
bond angles	°	bond angles	°	bond angles	°
O8-Cd2-O7#2	96.7(6)	O8-Cd1-O1	106.2(3)	O8-Cd2-O3	105.7(3)
O3-Cd2-O7#2	75.0(5)	O8-Cd1-O4#1	106.4(3)	O8-Cd2-N4	112.1(3)
N4-Cd2-O7#2	150.8(6)	O1-Cd1-O4#1	121.3(2)	O3-Cd2-N4	100.4(3)
O6-Cd2-O7#2	57.0(5)	O8-Cd1-N1	118.2(3)	O8-Cd2-O6	115.2(3)
O4#1-Cd1-N1	100.7(3)	N4-Cd2-O6	104.0(3)	O3-Cd2-O6	118.4(4)
O1-Cd1-N1	104.8(3)				

Symmetry codes:#1 x, y+1, z; #2 -x+1, -y, z+1/2.
